# The Elevated Serum Level of IFN-*γ* in Patients with Failed Back Surgery Syndrome Remains Unchanged after Spinal Cord Stimulation

**DOI:** 10.1155/2019/2606808

**Published:** 2019-01-14

**Authors:** Piotr Kamieniak, Joanna Bielewicz, Cezary Grochowski, Jakub Litak, Agnieszka Bojarska-Junak, Beata Daniluk, Tomasz Trojanowski

**Affiliations:** ^1^Department of Neurosurgery, Medical University of Lublin, Poland; ^2^Department of Neurology, Medical University of Lublin, Poland; ^3^Department of Clinical Immunology, Medical University of Lublin, Poland; ^4^Institute of Psychology, Marie Curie-Skłodowska University in Lublin, Poland

## Abstract

**Objectives:**

We investigated the influence of spinal cord stimulation (SCS) on IFN-*γ*, IL-1*β*, IL-6, TNF-*α*, IL-10, and TGF-*β* serum levels in failed back surgery syndrome (FBSS) patients. The study will try to give new insights into the mechanism of SCS action and the role of IFN-*γ* and other cytokines in neuropathic pain (NP) development.

**Materials and Methods:**

Clinical and biochemical assessment was conducted in four groups of patients: *group 0* consisted of 24 FBSS patients qualified to SCS therapy, *group 1* included 17 patients who were one month after implantation, *group 2* featured 12 patients who were 3 months after the implantation, and *group C* (the control group) with no NP. Clinical status was assessed with the use of Numeric Rating Scale (NRS), the Pain Rating Index of McGill Pain Questionnaire (SF-MPQ), the Oswestry Disability Index (ODI), and Beck Depression Inventory (BDI). The plasma concentrations of IFN-*γ* were ascertained by an immunoenzymatic method.

**Results:**

We found a significant difference between the patients before SCS and controls' serum level of IFN-*γ*. Similarly, a significantly higher level of TNF-*α* and significantly lower level of IL-10 in FBSS patients than controls were observed. The significant differences were not observed between SCS patients 3 months after the procedure and controls' serum level of IFN-*γ* and other cytokines. We noticed a positive correlation between IFN-*γ* concentration with NRS back value before SCS and positive correlation between IFN-*γ* concentration after SCS with NRS leg value before SCS. Higher IFN-*γ* concentrations accompanied higher NRS values. Levels of TGF-*β* and IL-10 may correlate with physical ability and depressive behavior.

**Conclusions:**

SCS did not influence serum cytokine levels significantly. Serum concentration of IFN-*γ* may be recognized as an occasional pain factor because of its significantly higher level in FBSS patients versus controls and higher IFN-*γ* value accompanying higher pain intensity.

## 1. Introduction

Failed back surgery syndrome (FBSS) is one of the unsolved treatment problems that occur after spinal surgery and it is also believed to be one of frequent causes of neuropathic pain. Patients with FBSS often suffer from epidural, intraneural, or perineural fibrosis and scar tissue. They usually do not respond to classic spinal surgery. The treatment of chronic neuropathic pain which results from FBSS is difficult to manage because of poor understanding of pain mechanisms and difficulties with objective assessment of pain intensity. Recently, spinal cord stimulation (SCS) improving quality of life and patient ability has been recognized as one of the most useful methods for neuropathic pain treatment [1, 2]. Detailed mechanisms of spinal cord stimulation activity are unclear. Pain stimuli are transmitted through nerve pathways in the dorsal horns of the spinal cord. The activation of myelinated A-fibres in the dorsal horns can block up pain transmission in the spinal cord [3, 4]. SCS may change extracellular gamma-aminobutyric acid (GABA) which leads to intracellular GABA reduction [5, 6]. Other studies suggest that SCS diminishes the overexcitability of wide dynamic range (WDR) neurons in the dorsal horn, activates the receptors of GABA, and increases the release of acetylcholine by acting on the M4 muscarine receptors [7].

The main role played in the formation and maintenance of neuropathic pain (NP) is nerve irritation and inflammation which produce changes in the serum expression of cytokines [8]. Cytokines are signaling molecules of the immune system. There are proinflammatory cytokines such as IFN-*γ*, TNF-*α*, IL-1*β*, and IL-6 associated with the presence of pain and anti-inflammatory cytokines such as TGF-*β* and IL-10 associated with pain relief [9–11]. Cytokines, produced by the immune and glial cells, are believed to modulate neuronal transmission and play a role in the balance between proinflammatory and anti-inflammatory factors producing neuropathic pain feeling. Anti-inflammatory cytokines, such as IL-10, are produced by activated macrophages and monocytes and may inhibit proinflammatory cytokine synthesis [12]. After nerve injury, proinflammatory cytokines such as TNF-*α* and IL-6 are upregulated in the animal models [13–20]. The administration of IL-1*β* produces pain behavior in rats [21, 22]. Parallelly, the IL-10 level decreases after nerve chronic constriction injury (CCI) and intrathecal administration of TGF-*β* reduces pain, secondary to CCI in rats [23, 24].

In our interest is especially IFN-*γ*. Proinflammatory cytokines such as IFN-*γ* produced by the immune and glial cells are believed to especially modulate neuronal transmission and play a role in neuropathic pain feeling. After nerve injury, IFN-*γ* is upregulated in the rat dorsal horn and cooperates in neuropathic pain induction and maintenance [25–27]. IFN-*γ* induces a long-lasting depolarization of inhibitory circuits sensitizing at the same time the ascending pain tracts [28]. Neuron-glia interaction plays an important role in induction of central sensitization [29, 30]. In vitro investigations have shown that IFN-*γ* may inhibit glutamate receptor 1- (GluR1-) positive interneurons in the dorsal horns of the spinal cord [28, 31, 32]. The IFN-*γ* receptor forms a special receptor complex with the AMPA receptor subunit GluR1 [32]. Both, proinflammatory and anti-inflammatory cytokines, are targets for the treatment of neuropathic pain. However, little is known about the role of IFN-*γ* and other cytokines in SCS.

The aim of this study is to observe if SCS influences analgesic response, IFN-*γ*, and other cytokine serum levels.

## 2. Materials and Methods

A total of 24 patients included in the study (11 men and 13 women, mean age 55,87 years, range 33-78 years) were admitted to the Department of Neurosurgery of Medical University of Lublin in Poland and underwent SCS for the management of pain resulting from FBSS. Magnetic resonance imaging was used to choose and verify patients with FBSS. Cases such as recurrent disc herniations, spinal stenosis or instability, spinal tumors, and infections were excluded. Coexisting neurologic and psychiatric diseases, systemic and inflammatory diseases, use of corticosteroids, tumors, active infections, severe depression, hypochondriac behavior, and alcoholic abuse belonged to the additional exclusion criteria.

### 2.1. Neurosurgical Procedure

The patients have been implanted with 16 electrodes of Medtronic equipment. The surgical operation was performed under general anesthesia. The implantation of paddle was performed by laminectomy at the Th10-Th12 level. The procedures consisted of two phases. The trial period usually lasted for 3 to 4 days with the use of the external trial stimulator. Then, the decision about implantation of an impulse generator (IPG) was undertaken when more than 50% pain reduction was obtained in the NRS. 17 patients, out of the 24 primary qualified, found SCS as enough satisfactory treatment. The opioids were used to ameliorate pain caused by the implantation procedure during the postoperative period (2-3 days). The SCS parameters were recorded which are shown in Table 1.

### 2.2. Clinical Assessment

All patients underwent standard physical and neurological examination three times: prior to surgery, one month after, and three months after the procedure. Motor deficits, reflexes dysfunctions, and straight leg-raising test were evaluated. Patients filled in a questionnaire about the intensity and duration of pain according to the Numeric Rating Scale (NRS), questionnaire about neuropathic pain by the Pain Rating Index of Short-Form McGill Pain Questionnaire (SF-MPQ), Oswestry Disability Index (ODI), and Beck Depression Inventory (BDI). The individual SCS parameters for each patient to achieve analgesia were recorded. The results were compared with outcomes of 24 healthy volunteers, used as controls, from the community hospital without previous back and sciatica pain. The 7 patients, out of the 24 primary qualified group, did not have implantation because of coexistence of medical conditions which excluded under general anesthesia, withdrawal of written consent for operation, or lack of efficacy of SCS during the trial period. The patients from group 1 were evaluated 1 month after the operation. The 5 patients missed the control visit so 12 patients from group 1 consisted in group 2, which was also evaluated after 3 months since operation. The characteristic of patient groups is presented in Table 2.

### 2.3. Measurement of Serum Cytokine Levels

Blood samples were collected just prior to surgery, one month after, and three months after. Five milliliters of venous blood was collected in the morning and immediately centrifuged at 4000 *g* for 15 minutes. The serum samples were frozen at -80°C until analyzed.

A commercial enzyme-linked immunosorbent assay (ELISA) kit (human IFN-*γ*, IL-1*β*, IL-6, TNF-*α*, IL-10, and TGF-*β* Quantikine ELISA Kit, R&D Systems) was used for a quantitative determination of human IFN-*γ* in plasma samples. We followed the protocol recommended by the manufacturer. The ELISA Reader Victor (PerkinElmer, USA) was used in the procedure. Samples were assayed in duplicate.

The study was approved by the Local Human Research Ethics Committee. All participating patients were included following the signature of the written informed consent statement.

### 2.4. Data Analysis

Statistical analysis was performed with the use of IBM SPSS Statistics (version 24.0). Data was reported as the minimum, maximum, and mean values as well as standard deviation. For intergroup comparisons, we used Student's *t*-test for two independent samples and nonparametric Mann-Whitney *U* test. To analyze differences between the cytokine level before and after surgery, we used the nonparametric Wilcoxon test and Student's *t*-test for dependent samples. Cohen's *d* and rank correlation coefficients (*r*_*g*_) were used to assess the effect size. The Spearman rank correlation coefficient (*r*_*s*_) was applied to assess the relationships between variables. The level of significance was *α* = 0, 05.

## 3. Results

Our research was conducted in 4 groups of patients. Group 0 consisted of 24 patients with neuropathic pain because of FBSS (FBSS group) within the mean age *M* = 55, 87 (SD = 11,55). Among them, there were 13 female (F) patients and 11 men (M), and 13 were physical workers. In the past, they were operated on several times, average 2,8 (min = 1, max = 10). The average time of pain duration before operation was *M* = 130 months (SD = 114). The patients of group 0 were assessed just before the performance of the procedure of SCS implantation. Group 1 consisted of 17 (8 F, 9M) of these patients who were qualified for implantation of SCS. They were with mean age *M* = 56, 24 (SD = 12, 7). The 59 percent of them worked as physical workers. The patients were operated on several times at an average of 3 times (min = 1, max = 10). The mean time of pain duration was 146 months (SD = 126). We also distinguished group 2 consisting of 12 patients (mean age *M* = 56, 69, SD = 14, 4; F = 5, M = 7) from group 1 which were additionally evaluated 3 months after operation. Group 2 was smaller than group 1 because 5 patients did not come for follow-up visit. The control group included 24 healthy volunteers from the community hospital without previous back and sciatica pain (see Table 2).

After SCS, we observed a significant decrease of back pain—before implantation, the mean NRS of the back = 6, 86 (SD = 2, 60); after 30 days, the mean NRS of the back = 3, 82 (SD = 2, 90) (*t*_10_ = 3,573; *p* = 0,003; effect size *d* = 0, 99); and after 90 days, the mean NRS of the back *M* = 2, 55 (SD = 2, 84) (*t*_10_ = 2,055; *p* = 0,033; effect size *d* = 0, 44). Similarly, we observed a significant decrease of lower limb pain—before SCS, the mean NRS of the leg *M* = 7, 12 (SD = 1, 96); after 30 days, the mean NRS of the leg *M* = 5, 38 (SD = 2, 02) (*t*_12_ = 2,465; *p* = 0,015; effect size *d* = 0, 92); and 90 days after, the mean NRS of the leg *M* = 3, 35; (SD = 2, 63), (*t*_10_ = 1,663; *p* = 0,063) (see Table 3).

The significant decrease in disability followed the SCS procedure; the index of disability is measured by the ODI scale (*t*_12_ = 2,263; *p* = 0,021, effect size *d* = 0, 80). Before the procedure, the mean ODI *M* = 31, 47 points (SD = 6, 23) and after 30 days, the ODI *M* = 26, 15 points (SD = 7, 87). The results after 90 days did not change significantly (*t*_12_ = 1,188; *p* = 0,131). Relationships between BDI scores were not significant (see Table 3).

We observed a significant difference between pain in patients before SCS (*M* = 13.61, SD = 7, 58) and control (*M* = 9, 44, SD = 4, 47) serum level of IFN-*γ* (*z* = −2,174; *p* = 0, 03; effect size *r*_*g*_ = 0, 46). Significant differences were not reported between SCS patients after 3 months and control serum level of IFN-*γ* (see Table 4).

### 3.1. Level of IFN-*γ* before and after SCS and Relations between the Level of IFN-*γ* and the Results of Clinical Scales

IFN-*γ* serum level was not significantly changed after stimulation. Before SCS, the mean INF-*γ* concentration was 13,61 (SD = 7, 58); after 30 days, the mean level was 14,33 (SD = 7, 99); and after 90 days, the mean level was 12,12 (SD = 6, 47) (group 0 vs. group 1 (*z* = −0,827; *p* = 0,408), group 1 vs. group 2 (*z* = −1, 49; *p* = 0,136) (see Table 4).

We noticed a positive correlation between IFN-*γ* concentration with NRS back value (*r*_*s*_ = 0,618; *p* = 0,025) before SCS and positive correlation between IFN-*γ* concentration after SCS with NRS leg value (*r*_*s*_ = 0,643; *p* = 0,007) before SCS. Higher IFN-*γ* concentrations accompanied higher NRS values (see Table 5).

### 3.2. The Levels of Cytokines in the FBSS Group and Comparisons with the Control Group

The comparison of cytokine levels in the group of patients with pain from FBSS (*n* = 24) and the control group (Student's parametric significance *t*-test for two averages and nonparametric Mann-Whitney's *U* test) showed a significant difference only in the IL-10 level (*t*_17,5_ = −3, 04; *p* = 0,007) and TNF-*α* level (*z* = −2.194; *p* = 0.028). In the FBSS group, the average level of IL-10 was *M* = 9.14 (SD = 4.69), while in the control group *M* = 17.87 (SD = 10.50). On the contrary, patients with FBSS had higher levels of TNF-*αM* = 71.49 (SD = 83.04) than those in the control group *M* = 28.08 (SD = 21.48), whereas the groups did not significantly differ in IL-1*β*, IL-6, and TGF-*β* levels. The relationship between the occurrence of neuropathic pain and the level of IL-10 is high and moderate with regard to the TNF-*α* levels (see Table 6).

### 3.3. Changes in the Levels of Cytokines Caused by SCS

No statistically significant changes caused by SCS were observed in the level of IL-1*β*, IL-6, IL-10, TNF-*α*, and TGF-*β*, both 1 month and 3 months after surgery, although the level of IL-1*β* and TNF-*α* decreased 1 month and 3 months after SCS and the level of TGF-*β* increased 1 month and 3 months after SCS. The values of IL-6 and IL-10 did not change significantly (see Table 7).

But interestingly, in the follow-up study, 1 month after SCS, significant differences in IL-1*β*, IL-10, and TGF-*β* levels were found compared to the values measured in the control group. The level of IL-1*β* in SCS patients was lower when compared to the control group (*z* = −2.569; *p* = 0.010), similarly to the level of IL-10 (*z* = −2.662; *p* = 0.008). A month after SCS, patients showed higher TGF-*β* levels than those in the control group (*z* = −2.398; *p* = 0.016).

Similar differences were noted in the measurements of interleukin values 3 months after SCS. The level of IL-1*β* and IL-10 in patients was still significantly lower (for IL-1*βz* = −2.587; *p* = 0.010; for IL-10 *z* = −3.416; *p* = 0.001), while the level of TGF was higher (*z* = −2.440; *p* = 0.015) (see Table 7).

### 3.4. Relations between the Levels of Cytokines and the Results of Clinical Scales

Correlations concerning TGF-*β* and clinical outcomes were the most interesting. The level of TGF-*β* before surgery correlates negatively with the NRS leg scale results obtained in the follow-up one month after SCS (*r*_*s*_ = −0.608; *p* = 0.027) and with the ODI scale results in the follow-up study 3 months after SCS (*r*_*s*_ = −0.633; *p* = 0.027)—a higher TGF-*β* level before surgery correlates with less pain in the leg area a month after SCS and better functioning at 3 months after SCS.

The level of TGF-*β*, measured 3 months after surgery, correlates negatively with the NRS leg scale results obtained in the follow-up study one month after SCS (*r*_*s*_ = −0.872; *p* < 0.001) and with the results of the ODI scale in the follow-up month after SCS (*r*_*s*_ = −0.767; *p* = 0.006) and 3 months after SCS (*r*_*s*_ = −0.77; *p* = 0.003)—a higher TGF-*β* level measured 3 months after SCS correlates with less pain in the leg area a month after SCS and better ability a month and 3 months after SCS.

The level of TGF-*β*, measured 3 months after surgery, correlates negatively with BDI depression scale scores obtained in the follow-up study a month after SCS (*r*_*s*_ = −0.634; *p* = 0.027)—higher intensity of depressive symptoms in BDI one month after surgery correlates with lower TGF-*β* level in measurement 3 months after SCS.

The level of IL-10, before surgery, correlates negatively with the results of the ODI scale obtained in the follow-up study 3 months after SCS (*r*_*s*_ = −0.608; *p* = 0.036) and the severity of depressive symptoms in the BDI scale 3 months after SCS (*r*_*s*_ = −0.786; *p* = 0.021)—a higher level of IL-10 before surgery correlates with lower ODI scores (better physical ability of the patient) and less severe depressive symptoms at 3 months after SCS.

There were no significant correlations between the TNF-*α* level and the results of clinical scales both before SCS and in subsequent follow-ups (after 1 month and after 3 months).

Correlations between the results of clinical scales and levels of IL-1*β* and IL-6 were nonconclusive.

## 4. Discussion

Since the 1980s, SCS therapy has been available for patients suffering from neuropathic pain related to FBSS [1, 33]. More than 20000 neurostimulators are implanted each year [2]. Recently, clinical measure of SCS efficacy has been based on physical examination, comparing pain intensity and physical ability before and after implantation. Although the decrease of pain in our study is similar to the results obtained in other studies, we should remember about limitations [1, 33, 34]. Such assessment is mainly a subjective point of view from a patient's perspective. Hence, we need to supplement clinical examination and add objective data. The data which predicts the success of therapy is considered to be especially valuable. So there were some trials to seek the concentrations of neuroimmune mediators. Kinfe et al. reported that systemic circulating anti-inflammatory IL-10 was significantly increased after the burst SCS with back pain reduction [35]. McCarthy et al. described that cerebrospinal fluid levels of VEGF correlate with reported pain and are reduced by SCS in patients with FBSS [36]. The necessity of lumbar puncture is serious disadvantage during pain monitoring. Another limitation is that we have not measured the concentration of cytokines which occur close to the spinal cord or in the CSF so we have no direct information about cytokine changes after SCS. The search for neuroimmune mediators in the serum may be safer and more useful for the monitoring of treatment. It is known that serum levels of proinflammatory cytokines such as IL-6 and TNF-*α* are significantly upregulated during chronic pain [37–39]. To our knowledge, the present study is rare which analyzes proinflammatory IFN-*γ* concentrations in the human serum before and after SCS treatment. There are not any significant differences between IFN-*γ* serum concentrations before and after SCS. Nevertheless, it is interesting that higher IFN-*γ* concentrations accompany higher NRS scores. There are numerous reports about spontaneous pain occurring as a side effect of IFN-*γ* therapy in oncology [40]. Interferon-gamma (IFN-*γ*) is a proinflammatory cytokine and important modulator of central and peripheral immune response and additionally plays a role in the pathogenesis of neuropathic pain [41]. It is very interesting that the IFN-*γ* serum level in FBSS patients is significantly higher than that in the control group, although Koch et al. reported no relationship between plasma IFN-*γ* and pain intensity [37]. Moen et al. described that IFN-*γ* may contribute to the pathogenesis of nociceptive activity and pain behavior in acute lumbar radicular pain [42]. Studies performed on animal model revealed, that IFN-*γ* plays an important role as one of the factors leading to central sensitization, which results in neuropathic pain. [43]. So the FBSS patients' IFN-*γ* serum level differs from controls, and it is important to seek possibilities of objective assessment of SCS action.

It is known that serum levels of proinflammatory cytokines such as IL-1*β*, IL-6, and TNF-*α* are significantly upregulated during chronic neuropathic pain and anti-inflammatory cytokines such as IL-10 are significantly downregulated during chronic neuropathic pain state.

It is very interesting that the TNF-*α* serum level in FBSS patients was significantly higher than that in the control group. So FBSS patients estimated that cytokine serum levels differ from controls. The role of TNF-*α* is complex. During NP induced by nerve injury, the TNF-*α* action as recognized acts at different levels of the nervous system. TNF-*α* plays a role at the site of nerve injury, at the dorsal root ganglion, at the dorsal horn of the spinal cord, and at the brain [44]. Many studies have shown that elevated TNF-*α* is found peripherally during nerve damage in CCI animal models of neuropathic pain [13–16]. The upregulation of proinflammatory cytokines such as IL-1*β*, IL-6, and TNF-*α* has been observed during induced discogenic pain in animal models [45]. Additionally, it is known that serum levels of proinflammatory cytokines such as TNF-*α* and IL-6 may be significantly upregulated during chronic pain [37–39]. A potential mechanism of the nociceptive action of TNF-*α* may involve TNF-*α*-apoptosis-caspase [46]. Membrane K^+^ ion conductance is increased in a non-voltage-gated fashion leading to neuronal hyperexcitability [47]. Additionally, the efficacy of TNF-*α* antagonists depends on the type of neuropathic pain and is less effective in rat disc herniation models than in diabetic origin [48, 49].

The value of IL-10 was significantly lower than in the control group and did not change after SCS. It was observed that the IL-10 level correlates negatively with ODI and BDI scores. So a higher level of IL-10 accompanies better physical ability and less depressive behavior. The serum level of anti-inflammatory cytokine such as IL-10 was lower in patients with complex regional pain syndrome (CRPS) than in the controls [50]. The IL-10 level decreases after nerve CCI in the animal model [23]. IL-10 injected into the site of nerve damage decreases the number of endoneurial TNF-*α*-expressing cells and attenuates thermal hypersensitivity after CCI [51].

There were no significant differences in cytokine serum concentrations in the FBSS group before and after SCS. However, IL-1*β*, after SCS, decreases significantly and TGF-*β* significantly increases when compared to the results in controls.

It was observed that the levels of IL-*β*, 1 and 3 months after SCS, were lower than those before SCS but not significantly. Nevertheless, IL-1*β* levels 1 and 3 months after SCS were significantly lower than in controls. Elevated levels of IL-1*β* have been found in rats with chronic neuropathic pain, both peripherally and in the brain scan [52–56]. The IL-1*β* administration induces pain behavior in rats [21, 22]. Possible mechanisms depend on activation of the dorsal root ganglion and increased spinal cord activity [57–59]. Moreover, IL-1*β* induces central cyclooxygenase-2 (COX-2) upregulation, which results in an increase in the synthesis of PGE-2 in spinal cord neurons and the brain [60].

The values of IL-6 in the FBSS group and controls did not differ significantly as the values of IL-6 after SCS. The IL-6 dual nature is known. It is perceived as proinflammatory and anti-inflammatory cytokine. There are several human studies with different results. Some studies showed elevated IL-6 in a patient with herniated discs [61, 62]. Animal studies show that IL-6 may affect the synaptic activity in the superficial spinal cord and promote heat hyperalgesia if injected intrathecally [63]. In another study, the authors found no association between IL-6 levels and symptoms in a patient with CRPS [64]. Moreover, IL-6 is also a potent stimulator of the hypothalamic-pituitary-adrenal axis as alone or with other cytokines [65].

The value of TGF-*β* increases after SCS but not significantly. It is interesting that the TGF-*β* serum level after SCS was significantly higher than in controls before SCS and 3 months after and correlates negatively with NRS leg, ODI, and BDI scores. A higher TGF-*β* level characterizes less leg pain, better physical ability, and less depressive behavior. Intrathecal administration of TGF-*β* reduces pain secondary to CCI in the animal model [24, 66]. It is known that delivery of bone marrow stromal cells into the spinal cord of mice induces TGF-*β* secretion reducing CCI-related neuropathic pain [67].

The search for neuroimmune mediators in the serum may be safer and more useful for the monitoring of treatment. Cytokines are signaling proteins mediating activation, differentiation, and proliferation of immune cells and may play a role in chronic pain threshold. Cytokine synthesis is prompt and their actions are often localized with a short half-life period [44]. Pivotal for assessment of SCS efficacy for the serum level are changes of IFN-*γ* and other cytokine serum levels before and after implantation as well as when compared to healthy controls. A limited group of patients results from the homogeneity of a group (only FBSS) and exclusion of patients with serious comorbidities. It is not possible to exclude that surgery procedure itself had any influence on observed changes [68]. A significant effect of SCS on IFN-*γ* and other cytokine serum levels is not obvious. Further explorations are needed to explain what is the real neuroimmune target for SCS therapy.

## 5. Conclusion

Balance between proinflammatory and anti-inflammatory cytokines may play a role in neuropathic pain during FBSS. SCS did not influence serum cytokine levels significantly. Our data supports an important role for IFN-*γ* and other cytokines as neuroimmune factors in the pathophysiology of neuropathic pain from FBSS. Serum concentration of IFN-*γ* may be recognized as an occasional pain factor—significantly higher level in FBSS patients versus controls and higher IFN-*γ* value accompanying higher pain intensity. Levels of TGF-*β* and IL-10 may correlate with physical ability and depressive behavior. This study research does not confirm directly the relationship between SCS and IFN-*γ* and other cytokine serum levels but adds additional clinical light for cytokine action in neuropathic pain in FBSS patients. Further investigations for SCS influence on serum cytokines are needed.

## Figures and Tables

**Table 1:** Characteristics of SCS parameters.

No.	Age	Gender	SCS amplitude (V)	SCS frequency (Hz)	SCS pulse width (*μ*s)
1	46	M	4.5 (0), 4.2 (1), 4.3 (2)	40 (0, 1 ,2)	180 (0, 1, 2)
2	47	F	1.8 (0), 2.0 (1), 2.1 (2)	60 (0, 1, 2)	200 (0), 210 (1, 2)
3	70	F	6.5 (0), 6.4 (1), 6.0 (2)	60 (0, 1, 2)	180 (0, 1, 2)
4	70	M	2.3 (0), 2.5 (1), 2.3 (2)	60 (0, 1, 2)	210 (0, 1, 2)
5	68	F	5.0 (0), 4.8 (1, 2)	60 (0), 40 (1), 60 (2)	210 (0), 200 (1, 2)
6	38	M	5.0 (0), 5.2 (1, 2)	40 (0, 1, 2)	320 (0, 1, 2)
7	47	M	3.2 (0), 3.2 (1), 3.4 (2)	40 (0, 1, 2)	240 (0, 1, 2)
8	33	F	3.0 (0), 3.0 (1), 3.2 (2)	60 (0, 1, 2)	240 (0, 1, 2)
9	78	M	2.6 (0), 2.6 (1, 2)	60 (0), 40 (1), 60 (0)	180 (0, 1, 2)
10	61	M	3.7 (0), 3.6 (1, 2)	40 (0, 1, 2)	270 (0, 1, 2)
11	54	F	7.0 (0), 7.1 (1), 6.8 (2)	60 (0, 1, 2)	320 (0, 1, 2)
12	67	M	2.8 (0), 2.6 (1), 2.8 (2)	80 (0, 1, 2)	270 (0, 1, 2)
13	60	F	1.9 (0), 2.2 (1)	60 (0, 1)	180 (0, 1)
14	58	M	6.6 (0), 6.7 (1)	40 (0, 1)	300 (0, 1)
15	43	F	3.5 (0), 3.0 (1)	60 (0, 1)	240 (0, 1)
16	65	M	5.3 (0, 1)	80 (0), 60 (1)	240 (0, 1)
17	69	F	4.2 (0), 4.1 (1)	40 (0, 1)	210 (0, 1)

F: female; M: male; SCS: spinal cord stimulation; (0): measurement 24 hours after implantation; (1): measurement 1 month after implantation; (2): measurement 3 months after implantation.

**Table 2:** Characteristics of patients and controls.

	Group 0 *n* = 24	Group 1 *n* = 17	Group 2 *n* = 12	Controls *n* = 24	Group 0 vs. controls *p* value
Age Mean ± SD	55, 87 ± 11, 55	56, 24 ± 12, 7	56, 58 ± 14, 4	56, 69 ± 10, 48	0,807
Gender					
Female (F)	F: 13	F: 8	F: 5	F: 12	0,772
Male (M)	M: 11	M: 9	M: 7	M: 12	
Number of operations Mean ± SD	2, 83 ± 2, 12	3, 41 ± 2, 24	3, 58 ± 2, 57	—	—
Time of pain Mean ± SD	130,29 ± 114,3	145,6 ± 126,0	170,18 ± 137,75	—	—

**Table 3:** Clinical response to SCS.

	Group 0 *n* = 24 (before)	Group 1 *n* = 17 (1 month after)	Group 2 *n* = 12 (3 months after)	Group 0 vs. group 1 *p* value	Group 1 vs. group 2 *p* value
NRS back Mean ± SD	6, 86 ± 2, 6	3, 82 ± 2, 9	2, 55 ± 2, 84	**0,003**	**0,033**
NRS legs Mean ± SD	7, 12 ± 1, 96	5, 38 ± 2, 02	3, 25 ± 2, 63	**0,015**	0,063
PRI Mean ± SD	22, 12 ± 10, 7	13, 08 ± 7, 88	9, 27 ± 10, 11	**0,042**	**0,041**
ODI Mean ± SD	31, 47 ± 6, 23	26, 15 ± 7, 87	22, 5 ± 7, 57	**0,021**	0,131
BDI Mean ± SD	15, 94 ± 7, 87	13, 43 ± 10, 97	7, 38 ± 9, 29	0,073	0,121

NRS: Numeric Rating Scale; PRI: the Pain Rating Index; ODI: the Oswestry Disability Index; BDI: Beck Depression Inventory; Student's *t*-test for dependent samples with *p* < 0.05 highlighted in bold.

**Table 4:** Controls versus IFN-*γ* and cytokine serum level response to SCS.

	Controls *n* = 24	Group 0 *n* = 24 (before)	Group 1 *n* = 17 (1 month after)	Group 2 *n* = 12 (3 months after)
IFN-*γ* (pg/ml) Mean ± SD	9, 44 ± 4, 47	13, 61 ± 7, 58	14, 13 ± 7, 99	12, 12 ± 8, 47
*p* for controls versus SCS		**p** = 0, 03	**p** = 0,036	*p* = 0,329

**Table 5:** Correlations between IFN-*γ* serum level and NRS.

	NRS back 0 (before) 6, 86 ± 2, 6	NRS leg 0 (before) 7, 12 ± 1, 96
IFN-*γ* (before) 13, 61 ± 7, 58 pg/ml	**0,618**	0,493
	**p** = 0,025	*p* = 0,052
IFN-*γ* (after 1 month) 12, 33 ± 7, 99 pg/ml	0,414	**0,643**
	*p* = 0,159	**p** = 0,007

Spearman's correlation coefficient *r*_*s*_ with *p* < 0.05 highlighted in bold.

**Table 6:** Serum cytokine levels in the FBSS group versus controls.

	Group 0 (FBSS) *n* = 24	Controls *n* = 24	*p* value	Effect size
	a	b	a–b	
IFN-*γ* (pg/ml) Mean ± SD	13.61 ± 7.58	9.44 ± 4.47	**0.03** ^∗^	r_g_ = 0.46
IL-1*β* (pg/ml) Mean ± SD	3.003 ± 2.64	4.21 ± 3.49	0.204	—
IL-6 (pg/ml) Mean ± SD	3.28 ± 3.37	2.94 ± 1, 82	0.279	—
IL-10 (pg/ml) Mean ± SD	9.14 ± 4.69	17.87 ± 10.50	**0.007** ^∗∗^	*d* = 1.17
TNF-*α* (pg/ml) Mean ± SD	71.49 ± 83.04	28.08 ± 21.48	**0.028** ^∗^	*r*_*g*_ = 0.42
TGF-*β* (pg/ml) Mean ± SD	46175.94 ± 31495.74	32862.15 ± 19837.48	0.152	—

^∗^Significant difference at the level of *p* < 0.05. ^∗∗^Significant difference at the level of *p* < 0.01. Mann-Whitney *U* test with *p* < 0.05 highlighted in bold.

**Table 7:** Serum cytokine levels before and after SCS.

	Group 0 *n* = 24 (before)	Group 1 *n* = 17 (1 month after)	Group 2 *n* = 12 (3 month after)	*p* value	*p* value
	a	b	c	a–b	b–c
IFN-*γ* (pg/ml) Mean ± SD	13.61 ± 7.58	14.13 ± 7.99	12.12 ± 8.47	0.408	0.136
IL-1*β* (pg/ml) Mean ± SD	3.003 ± 2.64	1.58 ± 2.29	1.17 ± 0.93	0.554	0.906
IL-6 (pg/ml) Mean ± SD	3.28 ± 3.37	3.64 ± 3.08	3.52 ± 3.29	0.093	0.239
IL-10 (pg/ml) Mean ± SD	9.14 ± 4.69	9.62 ± 5.11	6, 82 ± 2.42	0.653	0.272
TNF-*α* (pg/ml) Mean ± SD	71.49 ± 83.04	50.42 ± 48.13	50.21 ± 39.09	0.981	0.695
TGF-*β* (pg/ml) Mean ± SD	46175.9 ± 31495.74	54409.3 ± 24356.72	54520.6 ± 16848.84	0.463	0.638

## Data Availability

The data used to support the findings of this study are available from the corresponding author upon request.
